# Clean and Safe Drinking Water Systems *via* Metagenomics Data and Artificial Intelligence: State-of-the-Art and Future Perspective

**DOI:** 10.3389/fmicb.2022.832452

**Published:** 2022-05-05

**Authors:** Asala Mahajna, Inez J. T. Dinkla, Gert Jan W. Euverink, Karel J. Keesman, Bayu Jayawardhana

**Affiliations:** ^1^Wetsus – European Centre of Excellence for Sustainable Water Technology, Leeuwarden, Netherlands; ^2^Engineering and Technology Institute Groningen, University of Groningen, Groningen, Netherlands; ^3^Mathematical and Statistical Methods – Biometris, Wageningen University, Wageningen, Netherlands

**Keywords:** drinking water production, drinking water monitoring, high-throughput sequencing technology, metagenomics, machine learning, water distribution

## Abstract

The use of next-generation sequencing technologies in drinking water distribution systems (DWDS) has shed insight into the microbial communities’ composition, and interaction in the drinking water microbiome. For the past two decades, various studies have been conducted in which metagenomics data have been collected over extended periods and analyzed spatially and temporally to understand the dynamics of microbial communities in DWDS. In this literature review, we outline the findings which were reported in the literature on what kind of occupancy-abundance patterns are exhibited in the drinking water microbiome, how the drinking water microbiome dynamically evolves spatially and temporally in the distribution networks, how different microbial communities co-exist, and what kind of clusters exist in the drinking water ecosystem. While data analysis in the current literature concerns mainly with confirmatory and exploratory questions pertaining to the use of metagenomics data for the analysis of DWDS microbiome, we present also future perspectives and the potential role of artificial intelligence (AI) and mechanistic models to address the predictive and mechanistic questions. The integration of meta-omics, AI, and mechanistic models transcends metagenomics into functional metagenomics, enabling deterministic understanding and control of DWDS for clean and safe drinking water systems of the future.

## Introduction

The importance of access to clean water and sanitation has been recognized worldwide as one of the main themes in the UN Sustainable Development Goals. While developed nations have connected their population to the water network, access to safe and clean water poses a challenge to the water management authorities. The rapid depletion of groundwater and the contamination of surface water by industrial, agricultural, and urban waste streams have contributed to this problem. Sanitation and hygiene also rely heavily on adequate access to clean water for preventing and containing diseases to reduce the spread of pathogens and viruses (WHO, 2020). While the majority of drinking water bacteria is not dangerous for human health and is actually useful for the production of drinking water at the treatment plant, these organisms can cause unpleasant taste, odor, and turbidity of drinking water when present in excess (van Lieverloo et al., 2002; Vreeburg et al., 2004). Around 80% of customers’ complaints to the water utilities are about unwanted aesthetic aspects of drinking water that are generated during its distribution. These impaired aesthetics, which are a result of the uncontrolled growth of indigenous bacteria in particles, sediments, and biofilms in distribution pipelines might even include the presence of invertebrates in the water (Polychronopolous et al., 2003; Vreeburg and Boxall, 2007).

Uncontrolled growth of indigenous bacteria in water distribution systems results in microbially induced operational problems in distribution pipes which introduce significant investment and maintenance costs for water utilities (Allion et al., 2011). In the Netherlands alone, investment costs on distribution pipelines require approximately 50% of water utility investments (de Moel et al., 2006). For example, sulfate-reducers and iron-oxidizers cause bio-corrosion of cast-iron pipes (Sun et al., 2014), and the growth of bacteria to high numbers in the form of a biofilm cause fouling of concrete pipes In addition, the suspension of some of the bacteria which are attached to particles, sediments, or biofilms in distribution pipes can result in turbid or discolored water (Vreeburg et al., 2004). These bacteria are non-pathogenic and their excessive growth makes the water yellowish (Vreeburg and Boxall, 2007). Iron particles and manganese precipitates in water which are partially produced by bio-corrosion of iron pipes (Sun et al., 2014) or manganese-oxidizing or reducing organisms (Cerrato et al., 2010) cause water to be red or black colored (Seth et al., 2004). Other bacteria produce molecules affecting the taste and odor of water. For example, Actinomycetes produce Geosmin which is responsible for an earthy-muddy water taste (Srinivasan and Sorial, 2011), and sulfate-reducing or sulfur-oxidizing bacteria can enhance a sulfur-based odor (Scott and Pepper, 2010). On top of that, fungi, and yeast induce other aesthetic problem that has been recorded in drinking water systems. They negatively alter water odor and taste Protozoa and invertebrates such as worms (e.g., Annelida), crustaceans (e.g., Asellidae), or snails (e.g., Mollusca) have also been found in distribution systems (Christensen et al., 2011). As protozoa and invertebrates are at the top of the trophic chain, they indicate the presence of a high number of bacteria in water.

This uncontrolled growth of indigenous bacteria during water distribution can result in the exceedance of water quality regulatory guidelines (Sartory, 2004). The current regulation dictates that water treatment processes should yield drinking water that causes less than 1 infection per 10,000 people per year. However, continuous threats from newly emerging micro-pollutants and the risk of recontamination due to the growth of environmental pathogens in drinking water sources are still a concern. For instance, numerous pathogens which are opportunistic and hygienically threatening such as *Legionella pneumophila*, *Aeromonas hydrophila*, *Pseudomonas aeruginosa*, *Klebsiella pneumoniae*, *Mycobacteria*, and *Campylobacter* are able to grow at low nutrient concentrations in drinking water distribution systems and/or in household pipelines.

Limiting changes in the bacterial community during drinking water distribution and the prevention of uncontrolled growth up to high bacterial cell numbers and to the occurrence of unwanted microorganisms is done through removing carbon sources and nutrients, inactivating pathogenic organisms, removing chemical toxic compounds, and improving the transparency, taste, odor, and color of the water at the water treatment plant. Achieving high-quality drinking water that is biologically stable during transportation is done through physical, chemical, and biological processes such as dosing chlorine, aeration, ozonation, UV irradiation, active carbon filtrations, coagulation, flocculation, sedimentation steps, and/or rapid or slow sand filtration. The choice of which steps to apply to treat the water will depend on the source of the water and the initial water quality. After treatment, the water is transported *via* a pipeline system to the point of use or discharge. In this transportation process, residual organic material and microorganisms in the water may alter the quality of the water in this distribution system. The microbiological activity influences the chemical composition of the water and vice versa. The presence of organic material in water sustains the growth of microorganisms that form undesired biofilms and/or turbidity in the distribution system. The current removal of the organic material in the upstream purification steps aims to minimize regrowth but does not always result in biologically stable water. A balance between the efforts put in the removal and the risks for regrowth may be found in the specific quality of the organic material (Hijnen et al., 2014). However, detailed characteristics of the organic material are largely unknown, hampering the design of more effective treatment steps to produce biological stable water, i.e., water that does not support the growth of bacteria and other organisms in the distribution system.

While many countries around the world add disinfectant (such as chlorine, mono-chloramine, or chlorine dioxide) to drinking water as a secondary disinfection step, some European countries such as the Netherlands, Germany, Austria, and Switzerland use extensive treatment strategies which eliminate the bacterial growth supporting compounds (nutrients) in the water supplied to limit the potential regrowth in the distribution system. One disadvantage of using disinfectants in drinking water is that disinfectants react with organic compounds which results in the potential formation of carcinogenic by-products. Therefore, the concentrations of added disinfectants are kept to a minimum, with a higher risk of regrowth. Both methods are very effective at limiting bacterial growth in drinking water distribution systems. Yet, microbial changes in drinking water during distribution have been recorded in many countries. A more comprehensive overview of the drinking water distribution system microbiome is provided by Gomez and Aggarwal (2019).

This paper presents a review of recent advances in the monitoring, production, and distribution of drinking water using various -omics technologies. Firstly, the literature on microbial ecology in drinking water systems is revisited and various standard practices by water management authorities to monitor their activities are presented in “Microbiome in Water Systems” section. In “NGS Technology for Drinking Water Distribution Systems” section, the emergence of genetic sequencing technology as a new key-enabling water technology is discussed. This high throughput technology can shed light on microbial activities in much finer detail and allows us to understand the dynamics and various roles of microbial communities. This knowledge, through the employment of artificial intelligence and mechanistic models, can in turn be used to monitor and control the biological processes in drinking water systems as illustrated in “Artificial Intelligence Methods in DWDS” section.

## Microbiome in Water Systems

### Factors Affecting Drinking Water Microbial Ecology

The complexity of water in a DWDS, as a living aquatic ecosystem, is further enhanced by numerous aspects which are influencing the network of microbial interactions that exist in it during its distribution. Some of the aspects that influence bacterial growth during water distribution are: (1) the existence of the food chain, (2) concentration and type of nutrients, (3) type and concentration of residual disinfectant (if any), (4) microcosmic environmental conditions found in bulk water, sediment and/or biofilm, (5) system-wide environmental conditions (temperature, pH, etc.), (6) prevailing hydraulic condition and pipe materials, (7) and water residence time/water age (Prest et al., 2016).

### Assessment of Drinking Water Microbial Quality

Characterizing organic material in water and quantifying its growth-promoting properties for micro-organisms has been previously done using different methods. The assimilable organic carbon (AOC) method is based on the measurement of the growth of two pure bacterial strains in a pasteurized water sample. The biodegradable dissolved organic carbon (BDOC) method measures the uptake of dissolved organic carbon (DOC) by the autochthonous bacteria in a water sample, the liquid chromatography–organic carbon detection technique (LC-OCD) identifies and quantifies natural organic matter constituents in aquatic environments, and the biofilm formation rate (BFR) method quantifies the ability of water to promote the growth of bacteria into a biofilm. However, these methods are indicative tools and do not provide detailed characteristics of the organic material which subsequently hampers real-time monitoring of treatment processes and their optimization.

**Figure 1:**
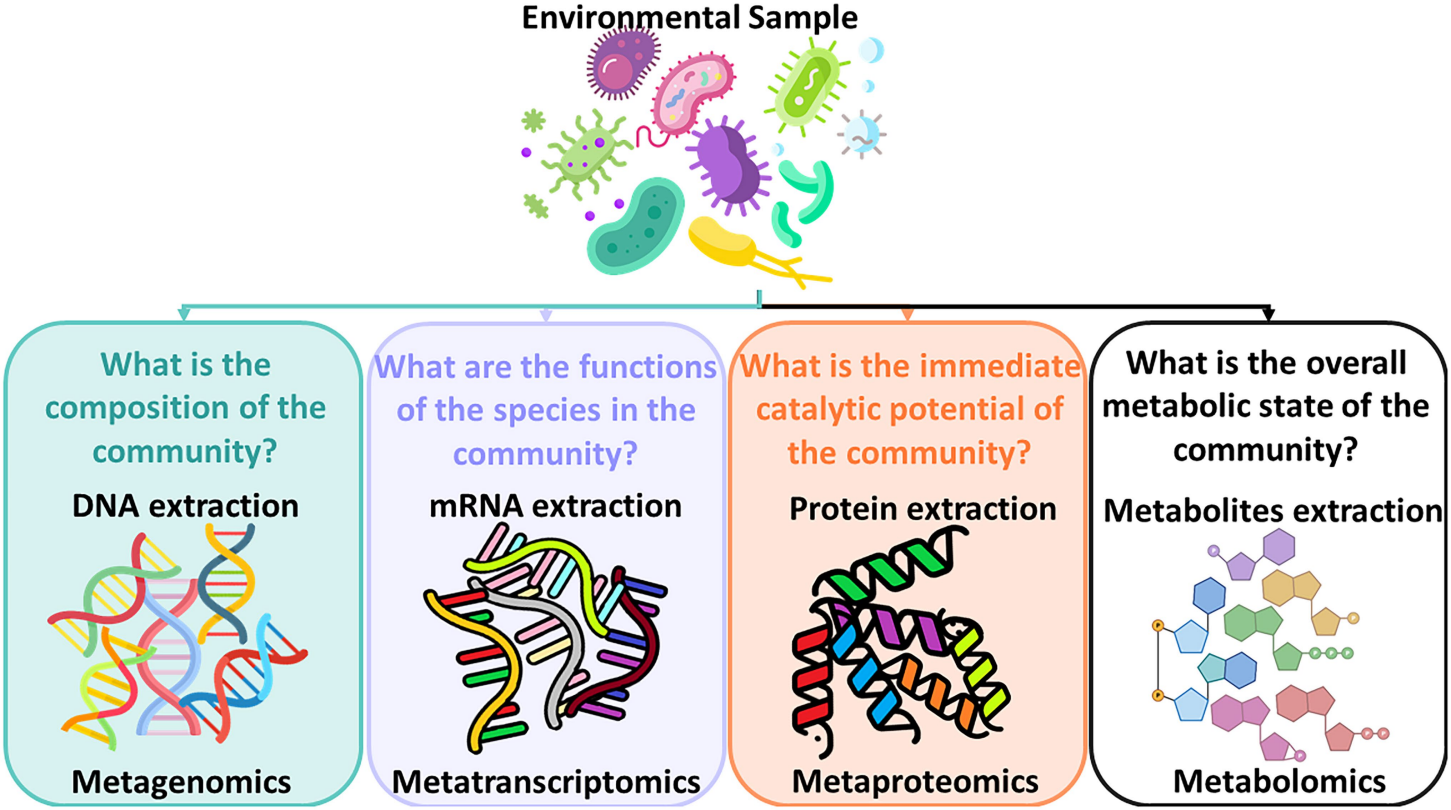
Subfields of meta-omics and the questions they address.

In addition, understanding microbial dynamics in drinking water distribution systems has been limited because of drawbacks of available methods for characterizing drinking water bacterial communities which rely heavily on culture-based techniques. Assessing water microbial quality has been traditionally done using heterotrophic plate counts (HPC) which is a method for bacterial enumeration. Alternatively, bioassays which are analytical methods for determining the concentration or potency of a substance by its effect on living cells or tissues can be applied. When microorganisms grow on organic substrates, specific degradation pathways are induced to enzymatically metabolize these organic compounds. Specific assays that can detect these enzymes require time-consuming, lengthy laboratory work. These methods are hypothesis-driven whose goal is to detect a targeted suspected compound and a selection of enzyme assays needs to be determined upfront. As these methods generate an assessment of the water quality with a time lag, detect only a minute fraction of the bacteria found in water in reality, and are limited when it comes to identifying all characteristics of the bacterial community found in the water, Next Generation Sequencing (NGS) technologies have been introduced in order to better assess the microbial drinking water quality. Initially, NGS technologies were utilized by the medical field for studying the gut microbiome (Malla et al., 2019) and by pharmaceutical industries for drug discoveries and personalized medicine (Vandeputte, 2021). Progressively, this technology has been introduced into the field of environmental microbiology to study soil microbiome (Nesme et al., 2016), and aquatic systems (Behera et al., 2021), and subsequently into the fields of wastewater treatment and drinking water quality and their respective processes (Tan et al., 2015; Zhang and Liu, 2019). While the development of the NGS technologies is a process of continuous enhancements (Slatko et al., 2018), the greatest advantage of NGS technologies is that they can provide a comprehensive assessment of the abundance, viability, and community composition of the microorganisms found in the water sample. The new field of meta-omics enables scientists to study mixtures of genetic material from all organisms in a sample. Figure 1 shows the subfields of meta-omics and what kind of questions these fields attempt to address.

### Mechanistic Models for Simulating Drinking Water Quality in Distribution Networks

Water utilities have been using mechanistic hydraulic models to simulate drinking water quality in drinking water distribution systems. These simulation tools are used for the purpose of optimizing the design of the water infrastructure and its facilities, the real-time hydraulic operation and monitoring of the network, simulation of events of contamination and tracing the source of such an event, and establishing guidelines for the operation and maintenance (O&M) of the supply system.

In 1990, the United States Environmental Protection Agency (USEPA) developed the Environmental Protection Agency Network (EPANET) which is the first computational software package for modeling the hydraulics of drinking water distribution systems (Rossman, 2000). Since then several commercially available spin-offs of EPANET were released. EPANET model start from a link-node structure where pipes are modelled as links, and junctions, hydraulic control elements, consumers, and sources are modelled as nodes. Drinking water quality is modeled in EPANET as an “additional simulation layer” on top of the hydraulic simulations which provide the core functionality of EPANET. Water age and source-tracing are two functionalities in EPANET which can provide an overarching assessment of the overall drinking water quality in distribution systems. Water age provides a proportional indicator of the decay of the residual disinfectant in the system and the formation of the respective disinfection by-products (DBP). On the other hand, source-tracing, which simulates the flow-path of water from the point of supply up to the point of consumption, has an added value when modeling drinking water quality in multi-quality water distribution systems where water comes from different sources. Source-tracing provides insight into a source of a contaminate in case of a contamination event, indicates potential mixing areas in the water supply network and provides knowledge about source influence areas in the system. Water age and source-tracing are mere high-level indicators of drinking water quality and in actuality drinking water quality may differ remarkably (Chenevey, 2022).

In EPANET, the Dynamic Water Quality Model (DWQM) serves as the basis for water quality modelling. For this, EPANET uses continuity equations for energy, mass, flow at nodes, flow for each storage component, mass for each storage component and each quality parameter, and equations for dilution requirements for modelling water quality under unsteady state flow conditions (Todini and Rossman, 2012). DWQM models single species concentration in the distribution system under first-order kinetics and plug-flow advection assumptions. However, the single species models do not account for microbial growth in the drinking water system and are merely limited to modeling process parameters throughout the distribution network (Woolschlager et al., 2005).

Recently, the National Health Systems Resource Centre (NHSRC) released a Multi-Species eXtension to EPANET called EPANET-MSX that enables modelling of numerous interacting species in the bulk flow and on the pipe walls, while modelling microbial growth, as well. This extension models heterotrophic microbial growth in both their fixed and suspended forms through solving a set of interdependent, multispecies, mass balance equations which is an expansion of the fundamental equations provided in the DWQM (Shang et al., 2011). Other multi-species models which are empirical, semi-mechanistic, and mechanistic were developed for research purposes to simulate microbial drinking water quality are not commercially available (). However, the modeling of microbial growth in the multi-species models is limited to two species/values (i.e., mass of free bacteria in bulk water, and mass of attached bacteria on pipe wall), and does not account for the rich microbial diversity which exist in the drinking water. In addition, the computation nature of EPANET-MSX, which solves a set of differential-algebraic equations (DAEs) in semi-explicit form, renders this model computationally inefficient for modeling the concentration of each bacterial species in a system that contains bacterial diversity in the magnitude of thousands. Hence, incorporating machine learning algorithms, which are good at handling data that are multi-dimensional and multi-variety, with metagenomics dataset can potentially present a computationally more efficient approach for simulating microbial drinking water quality (Rackauckas et al., 2020).

## NGS Technology for Drinking Water Distribution Systems

### Metagenomics Analysis for Microbial Communities

The emergence of new genetic sequencing technologies has enabled the gathering of crucial *in-situ* information related to microbial communities and occupancy-abundance dynamics in drinking water. In the pioneering work of Santo Domingo et al. in 2003 at the US Environmental Protection Agency Test and Evaluation (T&E) facility, metagenomics was applied to investigate the role of heterotrophic bacteria and ammonia-oxidizing bacteria in drinking water. They used a Distribution System Simulator (DSS) to assess the biofilm microbial composition in drinking water distribution systems (DWDS) due to the role of biofilms, which can contain human microbial pathogens, on public health. The researchers conducted 16S rDNA sequence analysis on both biofilm and bulk water samples from the DSS which revealed that *α-Proteobacteria* and *β-Proteobacteria* were the predominant bacteria in the feed water, discharge water, and biofilm samples. This early metagenomics application has been used to determine the effectiveness of disinfectant treatment to control microbial communities in DWDS. In 2005, Tokajian et al., conducted a phylogenetic assessment of heterotrophic bacteria using 16S rDNA sequencing from an operational water distribution system in Lebanon. Water samples were taken from raw unchlorinated aquifer water and from different sites in the distribution network on a bimonthly basis over a period of 1 year. The analysis confirmed the aforementioned observations (Santo Domingo et al., 2003; Williams et al., 2004) that the majority of bacteria in drinking water were α-, β-, and γ-Proteobacteria. In addition, the study also revealed a higher presence of *sphingomonads* in drinking water samples than reported elsewhere in literature which can be attributed to the specific operational conditions in Lebanon.

Once microbial communities are identified using metagenomics data, the next step is to establish their specific role, function, and interaction with the environment. In 2006, Eichler et al. used RNA- and DNA-based 16S rRNA gene fingerprinting further to gain a comprehensive understanding of how different factors (i.e., different raw water sources, different treatment processes, and distribution) influence the microbial communities in tap water designated for human consumption. Based on the DWDS of the city Braunschweig in Germany involving two water reservoirs with two different surface water types: oligotrophic water and dystrophic water, Eichler et al. (2006) observed that that major taxonomic groups typical of freshwaters such as *α-Proteobacteria*, *β-Proteobacteria*, and *Bacteroidetes* dominated the system. Comparative cluster analysis to the data revealed that there are three major types/clusters of communities in the system, each associated with the two types of surface water and to the chlorinated water, which is found to promote the growth of nitrifying bacteria. This work demonstrated the role of metagenomics analysis in revealing the importance of source water microflora to the drinking water microflora, in monitoring water quality, and in assessing the performance of different treatment processes. Further studies on the microbial diversity and composition in DWDS which support the metagenomic analysis in Eichler et al. (2006) were presented in Santo Domingo et al. (2003); Tokajian et al. (2005); Berney et al. (2009); Revetta et al. (2010), and Vital et al. (2012). The results of these studies are summarized in Supplementary Table 1.

### Metagenomics Analysis for Temporal and Spatial Distributions and Intra-community Dynamics

The first study to investigate spatial and temporal dynamics of drinking water microbiota using metagenomics was presented in Rudi et al. (2009). The authors used 16S rRNA sequencing analysis to assess temporal and spatial diversity of tap water (namely, kitchen tap and toilet tap) microbiota in a Norwegian hospital between January and July 2006 (for temporal analysis). In their study, the researchers used density distribution analyses to investigate tap-specific distributions of the bacterial groups. Based on the hierarchical clustering analysis, they concluded that the microbiota clustered according to the location (spatial) and not to the season (temporal). Related to a potential public health issue, metagenomics analysis in their study provided additional insights. It is shown in Rudi et al. (2009) that *Legionella* had the highest relative abundance for the pathogen-related bacteria in the dataset, especially in the low-usage tap, which can be investigated further for controlling local *Legionella* or other pathogens colonization. Such spatial metagenomics analysis can prevent pathogenic outbreaks from reoccurring, such as the well-known *Pseudomonas aeruginosa* outbreak in an intensive care unit at Akershus university hospital which could be traced back to a single tap.

In 2014, Pinto et al. (2014) used a spatially distributed and temporally varying sampling approach to conduct spatial–temporal surveying and occupancy-abundance modelling techniques using metagenomics analysis in a chlorinated drinking water distribution system in the USA. They sampled and analyzed the bacterial communities in water leaving the treatment plant from June 2010 to August 2011 at the clean water reservoir of a wastewater treatment plant and at three locations from three different sectors in the drinking water distribution system (resulting in nine locations in total). The analysis, which was based on total DNA extracts, resulted in the identification of 4,369 Operational Taxonomic Units (OTUs) at a 97% similarity cut-off, across 20 different phyla in the 138 water samples over the 15-month sampling period. In spite of the high diversity of the bacterial community found in the water, the *Proteobacteria* phylum is again the dominant DW bacterial community representing 60%–70% of the bacterial community for any given sample. Using Mantel’s test, changes in the microbial community can be explained by around 5% of the highly diverse OTUs which indicates that this subset of OTUs can be used to track changes in the community. For instance, it was observed that *β-* and *δ-Proteobacteria* dominated the DWDS during the summer months while *α-* and *γ-Proteobacteria* were dominant in the winter. *β*-Proteobacterium *Hydrogenophaga* (a genus of *comamonas* bacteria) in contrast displayed peak relative abundance in the colder months. Pinto et al. (2014) concluded also that biofilms in the neighborhood of each sampling location or possibly even microbial ingress into the DWDS led to the observed location-specific OTUs in the system.

Prest (2015) studied temporal dynamics in bacterial community characteristics during a 2-year drinking water monitoring campaign in a full-scale distribution system operating without detectable disinfectant residual. The data collected came from a total of 360 water samples which were sampled on a biweekly basis from Kralingen water treatment plant effluent and at one fixed location in the DWDS. The samples were analyzed for heterotrophic plate counts (HPC), *Aeromonas* plate counts, adenosine-tri-phosphate (ATP) concentrations, flow cytometric (FCM) total and intact cell counts (TCC, ICC), water temperature, pH, conductivity, total organic carbon (TOC) and assimilable organic carbon (AOC). Computational multivariate analyses showed that the change in microbial parameters between the water treatment plant and DWDS had a predictable annual trend comparable to the water seasonal temperature fluctuations and was negatively correlated to the AOC concentration in the water treatment plant effluent. Prest (2015) concluded that microbial growth in DWDS was not attributed to a single parameter only in the treated effluent. Roeselers et al. (2015) conducted a similar study in which spatial and temporal patterns in phylogenetic diversity were investigated using high-throughput sequencing technology in 32 DWDS networks in the Netherlands where residual disinfectant is not used. They observed that the microbial community compositions from water samples can be differentiated based on the source of the water sample, e.g., raw water and processed water in different locations. In addition, the researchers observed that community structures of processed water did not differ substantially from end-point tap water which indicates that network-specific communities are stable in time. The analysis on microbial community clusters showed that the treatment plant rather than the sampling time points differentiates drinking water microbial communities.

All of the above-mentioned findings were consistent with the conclusions made by Blokker et al. (2016) who used self-organizing maps for relating water quality and water age in DWDS from a multi-year Dutch and United Kingdom dataset. Their analysis showed that water age and temperature may be treated as independent parameters influencing microbial water quality. In addition, they concluded that there is a clear influence of temperature, which is dictated by seasonal change, on *Aeromonas* and the HPC at 22°C. They also showed that while water age has been traditionally used as a mathematical modelling tool to give an indication for all system-specific degradation of water quality, it appears to be of little value as an indicator for specific microbial water quality compared to water temperature. Their study recommends that specific DWDS conditions such as temperature, substrate concentration, and local shear stresses be incorporated in water quality models to better understand the risk of developing vulnerable water quality locations in drinking water distribution systems.

To assess the origin of bacteria in tap water and distribution system in an unchlorinated drinking water system, Liu et al. (2018) looked into the bacterial communities associated with biofilms, suspended particles, and loose deposits which are released in the distribution system as they are considered the major potential risk for drinking water bio-safety. They quantified the proportional contribution of the source water, treated water, and distribution system in determining the tap water bacterial community and concluded that the water purification process shaped the community of planktonic and suspended particle-associated bacteria in treated water. Correspondingly, Liu et al. (2018) recommended that tap water quality can be improved by both improving the purification steps and by cleaning the DWDS regularly.

In a recent study, Douterelo et al. (2018) used shotgun metagenomic sequencing to evaluate the taxonomic associations and functional aptitude of microbial communities found in chlorinated DWDS from two operational DWDS in the Southwest of the United Kingdom, where one DWDS is fed by surface water and the other one by groundwater. They isolated DNA from 24 samples which were taken from six bulk water and six biofilm samples at each sampling site. The shotgun metagenomic analysis showed that all domains of life (i.e., prokaryotes, eukaryotes, archaea, and viruses) are diversely present in the DWDS which is consistent with all previous metagenomics studies in DWDS. The researchers noted that the identification of metazoan DNA does not imply that the actual organisms are in the samples, but it can be used to indicate an ingress, e.g., free DNA released from animals or plants into the original source water or hydraulically introduced ingress. They concluded that limiting the entry of organic matter in the system can be an approach to inhibit the growth of biofilms in the system. Additionally, the researchers suggested that understanding the mechanism of biofilm formation can bring about the capacity to create the environmental conditions which favor the growth of infrastructure-protective extracellular polymeric substances (EPS) or exterminate pathogens. While the genus *Pseudomonas* has been used to indicate biofilm formation, they recommended the use of alternative bio-indicators of corrosion or biofilm formation in DWDS such as *Bacteroidetes*. Further studies on the microbial dynamics in DWDS which support the findings in the abovementioned studies are presented in Bae et al. (2019); Dai et al. (2019); Dias et al. (2019); Erdogan et al. (2019); Kori et al. (2019); Perrin et al. (2019); Brumfield et al. (2020); Maguvu et al. (2020); Siedlecka et al. (2020); Vavourakis et al. (2020); Atnafu et al. (2021); Bian et al. (2021); Kennedy et al. (2021), and Sevillano et al. (2021). A summary of the results of these studies is provided in Supplementary Table 1.

The aforementioned literature review has shown the applicability of metagenomics analysis to understand the role of spatial and temporal distribution and to study the dynamics of microbial communities in DWDS. A number of genetic markers can be identified for monitoring the variation in the communities that in turn provide the health status of DWDS. There are many ongoing research projects that are built on these findings allowing the development of monitoring systems using predictive models based on the variation in the relative abundance of genetic markers and on recent advances in data science, statistical learning, and artificial intelligence.

## Artificial Intelligence Methods in DWDS

### Current Lines of Enquiry on Microbial Dynamics

In previous sections, a literature overview has been presented on the use of metagenomics data which have been collected over extended periods and analyzed temporally and spatially to understand the dynamics of microbial communities in DWDS. These works addressed mostly confirmatory and exploratory questions corresponding to the use of metagenomics data for the analysis of DWDS. From a confirmatory angle, the results so far have addressed the questions on the associations between seasonality/location/type of source water/kind of disinfectant/treatment processes and different environmental parameters on the microbial community composition and structure found in DWDS. From an exploratory angle, research works hitherto have addressed the question of which factors influence most prevalently the microbial dynamics observed in DWDS. As the next step in data science, where data are used to answer predictive questions, there are currently many ongoing research activities where metagenomics data are analyzed for decision-making processes, such as process control and risk mitigation. These works involve the development of predictive models of DWDS that are enriched by real-time information of microbial communities’ activities from metagenomics data.

**Figure 2:**
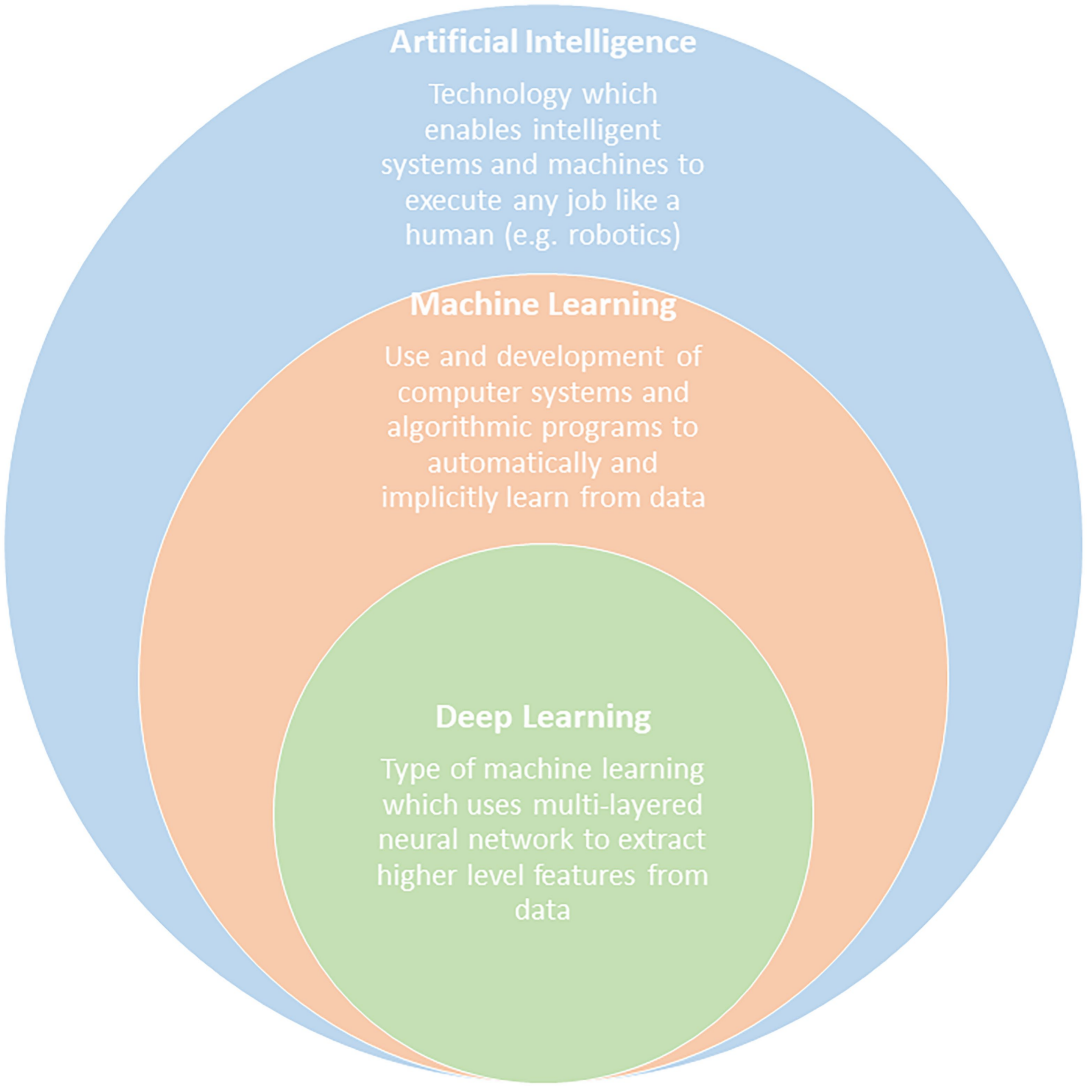
The circle of learning in artificial intelligence.

The field of machine learning, which is encompassed by the field of artificial intelligence (AI), can be used to process metagenomics data into meaningful information that can enrich predictive models of DWDS. Figure 2 shows the circles of learning methods within the AI field that incorporate recent advances in machine learning and deep learning. Based on the data structure, problem formulation, and the machine learning algorithm used, data science can address different aspects of control and optimization of DWDS and the quality monitoring thereof. In this regard, machine learning can be deployed for four categories of application in DWDS: modelling microbial network interactions, prediction and forecasting of microbial and chemical water quality, decision support for maintenance and operation, and system optimization.

Addressing any type of question using data for different applications can be done through the use of three main types of machine learning: unsupervised learning, supervised learning, and reinforced learning. Unsupervised machine learning algorithms aim to identify meaningful patterns in the data by looking for hidden features in the unlabeled dataset and inferring clusters, accordingly. The use of such algorithms to answer questions regarding prevalence clusters within the microbial communities of drinking water has been illustrated by Pinto et al. (2014) as mentioned above. K-means clustering, Neural Networks (NN), and Principal Component Analysis (PCA) are some of the unsupervised machine learning approaches which are used for solving clustering problems. Supervised machine learning algorithms are deployed on labeled training data sets to make predictions. Classification problems are problems where supervised machine learning algorithms can be used to predict which category something falls into. Naive Bayes Classifier, Support Vector Machines (SVM), Logistic Regression, and Neural Networks are some of the approaches that can be deployed to solve classification problems. In the DWDS case, Liu et al. (2018) used the Bayesian “SourceTracker” method to assess the origin of bacteria in tap water and distribution system. Supervised machine learning algorithms can also be used to solve regression problems, for instance, in making predictions on a continuous scale. Various regression methods (linear, nonlinear, or Bayesian) using nonlinear static, dynamic, or spatially distributed models, can be used in these cases. Negara et al. (2019) has used SVM to solve a nonlinear regression problem that maps metagenomics data from a waste-water treatment plant into the process parameters. Finally, reinforcement learning algorithms use feedback-based learning algorithms where actions and rewards are defined, involving the decision-making agent and environment, in order to maximize a given utility/value function.

### Knowledge Gaps and Latent Potential for the Discovery of Novel Lineages

In a study conducted by the United States Environmental Protection Agency (US EPA) which aimed at identifying microbial communities in drinking water by analyzing 16S rRNA-based clone libraries, the researcher found a majority mounting to 57.6% of the sequences belongs to the category of difficult-to-classify bacteria. The researchers observed that 44% of these difficult-to-classify sequences were closely related to sequences retrieved from preceding genomics-based drinking water studies. Thus, these hard-to-classify sequences are most likely indicative of novel lineages which are characteristic of the drinking water microbiome and may play vital roles in drinking water biogeochemical processes (Revetta et al., 2010). As a consequence of this knowledge gap, light must be shed on the limitations of any artificial-intelligence-based models that use metagenomics data because the performance of any data-driven mathematical model depends on the quality of data it is fed (Sessions and Valtorta, 2006; Alves et al., 2021; Sambasivan et al., 2021).

In their opinion paper Hull et al. (2019), highlight that research in the field of drinking water (DW) microbiome is lagging behind compared to research advancements in the fields of the human microbiome, and environmental microbiomes. Thus, they suggest that the field of DW microbiome can benefit greatly from combining efforts for building a DW microbe project (DWMP). By going in the footprints of other genome databases, the field of DW microbiome can benefit from enriching a central database to include within-species resolution data. In addition, further whole-genome sequencing of DW samples can tackle the issue of unclassified/unknown/sequences (Hull et al., 2019).

### Future Technology in DWDS: Meta-Transcriptomics

Meta-transcriptomics (RNA) data introduces additional dimensionality into the mathematical problem formulation that machine learning algorithms can accommodate to address questions regarding functionality. Meta-transcriptomics transcends metagenomics data analysis, where in addition to identifying the microbial communities in DWDS, it can provide information on the functions of each organism (functional metagenomics). One of the advantages of meta-transcriptomics is its ability to differentiate between the active part of a microbial community from the total community which can be quite distinct from one another. The extra knowledge on the functions of species in the microbial community in drinking water can provide valuable information for better understanding the metabolic pathways that are expressed in the bacteria that are present in the aquatic environment of drinking water. The information can be used by operators to deploy appropriate control actions that inhibit undesired metabolism and promote favorable ones (e.g., the metabolic pathways to convert major and minor carbon sources or specific compounds like pollutant degradation). Researchers in the medical field previously showed that meta-transcriptomics can provide a high-resolution picture of the microbiome’s functional dynamics (Lavelle and Sokol, 2018). From a meta-omics point of view, it is envisioned that meta-transcriptomics will be crucial for the next step in an obtaining accurate understanding of microbial communities’ activities in DWDS.

## Discussion

Metagenomics analysis of DWDS has revealed that high-resolution spatial and long-term temporal metagenomics data of DWDS provide insights on the variation of microbial communities under different environmental conditions. A group of genetic markers can subsequently be identified to monitor the dynamic changes in the drinking water microbiome. The ability to forecast the spatial distribution and temporal dynamics of a drinking water bacterial community can make water quality monitoring more cost-effective, contribute to public health safety by ensuring a safe water supply and increase the performance of process control strategies. Knowing the normal conditions for the operation of the system in its steady-state allows for finding anomalies and invasive pathogens faster. While in all the aforementioned literature (Supplementary Table 1), metagenomics data has been effectively collected over extended periods and analyzed to understand the dynamics of microbial water quality in both wastewater treatment plants and water distribution systems, the data analysis has been limited to correlation analysis of available process data. An integrated approach that combines the meta-genomic data with predictive kinetic-mechanistic modelling, potentially combined with machine learning techniques, is still lacking. Consequently, current and future research directions should aim towards the development of a new approach using machine learning techniques to interpret DNA and RNA Next Generation Sequencing (NGS) data in combination with chemical and physical process knowledge to form the basis of a deeper understanding and prediction of the biological and chemical processes in the DWDS. It will transcend metagenomics into functional metagenomics in the drinking water management systems.

## Author Contributions

ID, G-JE, KK, and BJ contributed to the ideation, design, review, and editing of the literature review paper. AM wrote the manuscript and contributed to the ideation and the design of the paper. All authors contributed to the article and approved the submitted version.

## Funding

The work is co-funded by the Dutch Ministry of Economic Affairs and Climate Policy, the European Union Regional Development Fund, the Province of Fryslân, and the Northern Netherlands Provinces.

## Conflict of Interest

The authors declare that the research was conducted in the absence of any commercial or financial relationships that could be construed as a potential conflict of interest.

## Publisher’s Note

All claims expressed in this article are solely those of the authors and do not necessarily represent those of their affiliated organizations, or those of the publisher, the editors and the reviewers. Any product that may be evaluated in this article, or claim that may be made by its manufacturer, is not guaranteed or endorsed by the publisher.
