# Supplementary material for: Clean and Safe Drinking Water Systems via Metagenomics Data and Artificial Intelligence: State-of-the-Art and Future Perspective
Source: Front Microbiol. 2022 May 5;13:832452. doi: 10.3389/fmicb.2022.832452 (PMC9121918; doi:10.3389/fmicb.2022.832452)
Supplement: Supplementary file 1 [file Table_1.pdf]

Supplementary Material

1 Supplementary Table

Reference	Data	Method	Conclusion
(Santo Domingo et al. 2003)	Biofilm and water samples were collected from the Distribution System Simulator (DSS) at the US Environmental Protection Agency Test and Evaluation (T&E) facility under six different disinfection regimes of chlorination and chloramination which were applied to the system for a total period of 36 weeks.	Metagenomics of Heterotrophic Bacteria and ammonia-oxidizing bacteria using 16S rDNA analysis	• *α-Proteobacteria* and *β-Proteobacteria* were the predominant bacteria in the feed water, discharge water, and biofilm samples.<br>• Bulk and biofilm bacterial communities differ on both levels of total community structure and heterotrophic population dynamics.<br>• The research suggests that type of disinfection treatments could influence the type of bacterial community inhabiting water distribution systems.
(Hoefel et al. 2003)	Samples of raw and potable waters taken from various locations around South Australia	Total cell count using flow cytometry of cultured bacteria	• Rapid technologies for bacteria enumeration have the potential to replace time-consuming culture-based techniques for the bacteriological assessment of water bacterial communities
(M. M. Williams 2004)	Three water samples were collected at each of the feed and discharge locations of the Cincinnati Distribution System Simulator (DSS) at the US Environmental Protection Agency Test and Evaluation (T&E) facility. The	Metagenomics of heterotrophic bacteria using 16S rDNA analysis	• *α-Proteobacteria, β-Proteobacteria, and γ-Proteobacteria* were the predominant bacteria in the feed water, and discharge water samples.<br>• Observations from this study suggest that bacteria from the *α-Proteobacteria* class are well-adapted to survive in drinking water distribution systems as they were the predominant phylogenetic class in all samples analyzed

	free-chlorine residual was amended with NH_3 to create a monochloramine residual in the supplied water in the DSS.		following either of the two disinfectant treatments (chlorination or mono-chlorination).
(Tokajian et al. 2005)	Water samples were taken from a raw unchlorinated underground source and from different sites in the distribution network on a bimonthly basis over a period of 1 year.	Metagenomics of heterotrophic bacteria using 16S rDNA analysis	• The major Gram-positive heterotrophic bacteria in drinking water were α-, β-, and γ-Proteobacteria.<br>• The study also revealed that the presence of different bacterial community compositions and structures can also be a result of different hydraulic operating conditions.
(Martiny et al. 2005)	Water samples were taken from both biofilm and bulk water from a non-chlorinated model drinking water distribution system operated under turbulent flow conditions	Metagenomics of cultivated bacteria using 16S rRNA analysis	• The researchers in this study observed abundance of nitrite-oxidizing bacteria.<br>• They infer that due to the low assimilable organic carbo concentration in the water, nitrite plays an important role as a substrate in the system.<br>• Their findings also suggest that while diverse microbial communities could possibly prevent elevated nitrite concentrations, they are adversely capable of depleting residual disinfectants added to the systems.
(Eichler et al. 2006)	Different samples of raw water and treated water were analyzed from the DWSS of the city of Braunschweig, Germany at the two reservoirs and 2 locations at the DWDS including a tap water location.	Metagenomics: RNA-and DNA-Based 16S rRNA Gene Fingerprinting	• Metagenomics analysis reveals that microbial community composition and structure in drinking water is dictated by the microflora of the source water.<br>• Chlorination causes a significant shift in the microbial community composition and structure.<br>• Chlorination may be introducing nitrifying bacteria to the drinking water supplies.

(Berney et al. 2009)	Three different types of non-chlorinated drinking water were<br><br>analyzed in this study, namely non-chlorinated household tap<br><br>water at 3 locations, 3 public drinking fountains, and 3 brands of bottled water.<br><br>The different water types were collected on separate days over a period of two months in Switzerland. Three separate samples were collected on the same day and analyzed immediately for each water type.	Total cell count using flow cytometry	• This study recommends the use of a combination of fluorescent staining, flow cytometry, and total ATP measurements to assess microbial viability in water as a monitoring tool and for further study of microbial processes.
(Poitelon et al. 2009)	Chlorinated drinking water from three different water treatment plants was sampled and analyzed. The treatment plants are supplied with surface water from nearby rivers.	Metagenomics: 16S rDNA analysis using serial analysis of V6 ribosomal sequence tag (SARST-V6) method.	• *α-Proteobacteria, β-Proteobacteria*, ***γ****-Proteobacteria*, and δ *-Proteobacteria* were the predominant bacteria in the finished drinking water.<br>• The observations in this study suggest that drinking water may contain higher diversity in microbial communities than reported in previous studies.
(Rudi et al. 2009)	Samples were taken from two hospital taps (kitchen and toilet) supplied from the same water source in the summer and the winter	Metagenomics: 16S rRNA sequencing analysis	After deploying Hierarchical clustering for the 1x1 OTU density distribution, the researchers found that the microbiota clustered according to tap and not the season.<br>• Legionella showed the highest relative abundance for the pathogen-related bacteria, especially in the low-usage tap. Thus, the researchers recommend investigating whether the

	(in January and July 2006) to compare the microbiota.		processes for local Legionella colonization can be related to tap usage. Understanding the ecological factors impacting Legionella and other pathogens is of tremendous importance for human health. This was exemplified at the Akershus University Hospital, where a Pseudomonas Aeruginosa outbreak in an intensive care unit could have been traced back to a single tap.
(Revetta et al. 2010)	Drinking water was collected from the tap in the laboratory at 12 different times during the summer months: June, August, and September.	Metagenomics: 16S rRNA sequencing analysis	• The researchers conclude that drinking water contains a diverse and dynamic microbial community as they observed that while the overall diversity is similar amongst the different samples, compositional memberships change with respect to time.<br>• The study suggests that the drinking water microbial community may contain novel bacterial clades because the researchers observed that a substantial portion of the viable bacteria in the samples are difficult-to-classify.
(Henne et al. 2012)	Bulk water and biofilm samples were collected from a chlorinated drinking water distribution network in Germany which is supplied with water from two surface water reservoirs (oligotrophic and dystrophic water).<br><br>Bulk water was sampled on the 23rd and 24th of June 2009 from several taps distributed around the campus, and from 2 households of the inner city of Braunschweig. Simultaneously, biofilm samples were collected as well. Due to construction works at the campus which resulted in the dismantling of the building, additional biofilm	Metagenomics: 16S rRNA sequencing analysis	• The researchers report observing both structure and composition of the bacterial core community in the bulk water was significantly comparable across the city, while all biofilm samples contained a distinctive community with no overlapping phylotypes from bulk water.<br>• All biofilm communities displayed higher relative abundances of single phylotypes and lower richness compared to bulk water.<br>• The researchers hypothesize that the better protection from the oxidative stress of the biofilm structure contribute to a higher fraction of active bacterial phylotypes and subsequently to a spatial similarity in the biofilm found in the drinking water distribution system as they observed that only biofilm communities sampled at nearby sampling points showed similar communities irrespective of support materials.

	samples were obtained on 7 and 14 May 2009.		
(Vital et al. 2012)	This study investigated microbial water quality from two water treatment plants and a water distribution network that is fed by the two treatment plants. The sources of water are surface water: river, and polder seepage water in the Netherlands. Samples were collected after each treatment step in both treatment plants and from 5 selected locations of the water distribution network on three separate days in August 2010.	Total cell count using flow cytometry	• The researchers reported that flow cytometry enabled a more detailed insight into microbial drinking water quality than conventional cultivation-based methods which only detect a minute fraction of intact cells in water.<br>• Conventional cultivation-based methods detect only 0.04% - 5.99% (depending on the cultivation method) of the intact cells which flow cytometry detects.
(McCoy and VanBriesen 2012)	Water samples were collected from a drinking water distribution system over two different years from Pittsburgh Water and Sewer Authority (PWSA). In the first year, seasonal samples were collected from fall 2016 to summer 2017.<br><br>Monthly samples were collected the following year from September 2008 to August 2009.<br><br>A sample in February 2009 was not collected.	Metagenomics: 16S<br><br>rRNA gene clone library analysis	• *α-Proteobacteria, β-Proteobacteria, and γ-Proteobacteria* classes are the core community in the drinking water distribution network.<br>• Seasonal fluctuations in relative populations were documented with decreased *α-proteobacteria* and increased *β-proteobacteria* diversity in the winter season compared to the other seasonal samples. This can be due to variations in chlorine dosing.<br>• This research suggests that changes in bacterial class distributions may be useful indicators of system disruptions as it was observed that the system has a core microbial community which has a seasonal pattern.

(Hwang et al. 2012)	To examine how different post-disinfection methods (i.e. chlorination and chloramination) influence drinking water microbial communities in an urban drinking water distribution system which is fed with groundwater as the source water and distributes water to an urban area with a pollution size of 40,000 persons, a study which used data from two years was conducted.<br><br>Data was collected in a way that insures that sampling dates coincided with the periods during which transitions from chloramines to free chlorine was applied by the water utility.<br><br>Water samples were in March 2010, January 2011, May 2010, July 2011, October 2010 and 2011.	Metagenomics: 16S rRNA gene sequencing analysis	• In order to explain the variations in microbial community profiles, the researchers deployed Nonmetric multidimensional scaling and canonical correspondence analysis. The researchers observed that clustering of samples based on disinfection types (free chlorine versus combined chlorine) and sampling time were observed to correlate to the shifts in microbial communities.<br>• However, the researchers also observed that the sampling location and water age had no detectable influence on the microbial community structure from measurement points at varying times.<br>• In addition, the researchers observed that major core populations that are *Cyanobacteria*, *Methylobacteriaceae*, *Sphingomonadaceae*, and *Xanthomonadaceae* were more abundant in chlorinated water, and *Methylophilaceae*, *Methylococcaceae*, and *Pseudomonadaceae* were more frequently present in chloraminated water.<br>• The researchers conclude that the data suggest that chloramines disinfection creates aquatic environmental conditions in which stronger selection of microbial populations occurs compared to the effects of chlorine disinfection. This conclusion is based on the fact that reversible shifts in microbial communities were especially pronounced with chloramination.
(Lautenschlager et al. 2013)	Water samples were collected from an unchlorinated water distribution network in Zurich. Water samples were taken from the reservoir and six points of the connected distribution network. The water samples were collected 6 times in the time period from November	Intact and total cell counts using flow cytometry and Metagenomics analysis using 16S rRNA gene	• The researcher deployed a multi-parametric approach that encompasses different aspects of microbial water quality (e.g. microbial growth potential, microbial abundance, and microbial community composition) to assess and continuously monitor biological stability in drinking water of the non-chlorinated distribution system of Zurich. The researchers report that microbial water quality in the system stayed stable over extended time periods with respect to intact bacterial cell concentrations, ATP measurements, and microbial communities.

	2008 until February 2009 and once in September 2010.	sequencing analysis	• Overall, the researcher recommends using a combination of multiple tools to assess microbial water quality as molecular methods can provide detailed information on the changes of microbial composition occurring in the system and subsequently contribute to an in-depth understanding of bio-stability during drinking water distribution than what was previously possible.
(McCoy and Vanbriesen 2014)	The chlorinated Pittsburgh Water and Sewer Authority (PWSA) drinking water distribution system was studied over one year to analyze drinking water bacterial diversity from temporal and spatial stand points. Bulk water samples were collected on a monthly basis from three different locations in the DWDS, from two taps, and from one continual flow pipe location in the system.	Metagenomics: 16S rRNA gene sequencing analysis	• The researchers observed that *α-Proteobacteria* displayed a positive relationship with the hypochlorite dose which varied in a seasonal manner. This observation explained the temporal bacterial diversity variability asseasonally-driven and resulting due to process control actions namely variations in chlorine concentration.<br>• The findings of this research reveal that samples clustered by months more than by locations which highlight the importance of temporal variability over spatial variability.<br>• In addition, Analysis of variance (ANOVA) of *α*-, *β*-, and ***γ**-Proteobacteria* and bacterial quantities for all collected samples indicate that temporal patterns are more significant than spatial patterns in this system environment.
(Liu et al. 2014)	Samples were collected from unchlorinated DWDS in The Netherlands. Three sites were selected from the distribution system that had not been flushed since their construction in 1966/1981/1984.	Intact and total cell counts using flow cytometry and Metagenomics analysis using 16S rRNA gene sequencing analysis	• The researchers observed that while bacterial communities in bulk water, pipe wall biofilm, and suspended solids throughout the distribution system are relatively stable, the bacterial communities present in loose deposits were dependent on the amount of loose deposits locally.<br>• In addition, the researchers observed that the bulk water bacteria which was dominated by *Polaromonas* were clearly different from the bacteria from the other three phases which were dominated by *Sphingomonas*. This observation indicates that bacteria within the phases of suspended solids, loose deposits, and pipe wall biofilm were similar in phylogenetic composition.<br>• Loose deposits are characterized by a protection layer for bacteria from disinfectants and hydraulic mobility which

			render them as vital agents in the pathogenic spread and threats to public health in the medium of drinking water. Thus, the researchers in this study highlight the importance of integrating knowledge from all of the four phases of microbial growth in DWDS (i.e. bulk water, pipe wall biofilm, loose deposits, and suspended solids) into modeling DWDS microbial ecology.
(Pinto et al. 2014)	In an attempt to understand bacterial dynamics in the chlorinated drinking water distribution system of Ann Arbor, Michigan, USA. The researchers conducted a 15-month long spatial-temporal survey. 150 samples were collected on three consecutive days on a monthly basis from June 2010 to August 2011. The samples were collected from the reservoir at the DWTP immediately before the treated water was pumped into the DWDS and at nine different sampling locations in three sectors of DWDS.	Metagenomics analysis using 16S rRNA gene sequencing analysis	• From the data gathered from the 15-month long spatial-temporal survey, and subsequent occupancy-abundance analysis the researchers report that the bacterial community spatial dynamics of distance decay and dispersivity conform to the layout of the drinking water distribution system.<br>• In addition, the researchers report that the patterns in temporal trends were stronger than those for the spatial dynamics.<br>• The data exhibited annual reproducibility – this demonstrated cyclical seasonality which correlated with temperature and source water use patterns.<br>• After conducting networks analysis, the researchers observed that two seasonal bacterial clusters consisting of multiple taxa with different networks of association within the larger drinking water bacterial community were the main drivers of the temporal trends.<br>• The study recommends establishing long-term microbial observatories that collect high-resolution, spatially distributed, multiyear time series of community composition and environmental variables to advance the development and validation of the predictive framework for drinking water microbial quality.
(Prest et al. 2014)	Samples were taken at the treatment outlet of the drinking water treatment plant and at one location in the network. Samples were	Intact and total cell counts using flow cytometry and	• Separately and congruently, FCM and pyrosequencing results revealed that the microbial community altered during distribution, which was not discovered with conventional monitoring techniques.

	collected from the two locations every 5 min for 1 h. To evaluate variations in water quality on short time scales (i.e. morning vs. afternoon), this procedure was performed in the morning from 08:00 to 09:00 and repeated in the afternoon of the same day from 13:00 to 14:00.<br><br>In sum, 52 bulk water samples were analyzed with FCM, pyrosequencing<br><br>and conventional methods (adenosine-triphosphate, ATP; heterotrophic plate count, HPC).	Metagenomics analysis using 16S rRNA gene	• In an attempt to evaluate and assess microbial changes in drinking water distribution systems, the researcher employed a methodology that combines flow cytometry (FCM) and 16S rRNA gene pyrosequencing data, the results revealed an increase in cell concentrations of specific bacterial phyla (e.g., *Proteobacteria*), along with a decrease in other phyla (e.g., *Actinobacteria*). This observation couldn't be possibly made by using one of the methods individually.<br>• The researchers report that the combination of FCM and pyrosequencing methods is a promising approach for future drinking water microbial quality monitoring and for advanced studies on drinking water network ecology.
(Huang et al. 2014)	Water was simultaneously sampled after sand filtration, Chlorine dosing, and after distribution from the tap. The sampling was repeated four times at each sampling campaign during August, September, October, and November of 2011.	Metagenomics analysis using 16S rRNA gene	• The researchers used 454 pyrosequencing and Illumina high-throughput sequencing in order to comprehensively investigate bacterial virulence in drinking water.<br>• The researchers observed that both diversity and abundance of pathogenic bacteria decreased after the chlorination and increased after the pipeline distribution.<br>• The researchers report that while many pathogens disappeared after chlorine disinfection, *P. Aeruginosa* and *Leptospira Interrogans* were still detected in the tap water.
(Roeselers et al. 2015)	In this study, the researchers looked at the microbial drinking water quality in an unchlorinated system before treatment, after treatment, and after distribution. The study was conducted in The Netherlands and the samples were collected	Metagenomics analysis using 16S rRNA gene	• The researchers observed that the microbial water quality of raw source water dictated system-specific microbiome including distinctive treated water microbial quality, and distributed water quality in the supply network.<br>• In addition, the researchers report that the treatment process significantly altered the microbial water quality. They report that in each network major differences in community

	from 32 different water treatment plants which are fed with groundwater, and the supplied water was sampled from the four different water distribution networks which are connected to these water treatment plants. In total, the researchers analyzed 154 water samples. The samples from the respective networks were sampled at three different time points within a period of 17 months (May-July 2012, March 2013, and September 2013).		compositions were observed between raw and processed water, while community structures of processed water did not change substantially from end-point tap water.<br>• In addition, the researchers observed that network-specific microbial communities were surprisingly stable in time and that end-point water samples within the same distribution network had very similar community structures.
(El-Chakhtoura et al. 2015)	156 samples were collected from a non-chlorinated water distribution system in The Netherlands at a high temporal resolution. The samples were collected from the outlet of a treatment plant and at one location in the connected water distribution network. The source water is surface water from the Meuse River.<br><br>The researchers studied the data at an hour, day, and week time scales. Hence, respective to the time-scale analyzed, the researchers deployed different sampling patterns.	Intact and total cell counts using flow cytometry and Metagenomics analysis using 16S rRNA gene	• The researchers report that bacterial community profile at the treatment plant and distribution network locations stayed relatively constant throughout different time-scale analytics.<br>• From a spatial perspective, the researchers report that the bacterial community structure changed during distribution while observing greater bacterial richness detected in the network.<br>• In addition, the researchers report that the treatment strategy shapes the microbiome in the system where a substantial core microbiome was shared between the produced and distributed water.<br>• Finally, this study reports that rare taxa which exhibited the highest dynamicity are causing major change detected during water distribution.<br>• The researchers suggest that with the advance of more accurate microbial measurement and assessment tools, the concept of biological stability needs to be revised.

(Bautista-De los Santos et al. 2016)	In this study, the researcher used 14 datasets that were either publicly available or made available upon data request from previous studies conducted in the USA, China, Netherlands, UK, Switzerland, Australia, and France.	Metagenomics analysis using 16S rRNA gene	• In this study, the researchers report that *Proteobacteria* dominate drinking water bacterial communities irrespective of the origin of the dataset and whether disinfectant is present or absent, and regardless of disinfectant residual type.<br>• Microbial communities in systems with disinfectant residuals are less diverse than in those which are free of disinfectant residuals.<br>• The researchers encourage the scientific community to archive the outcomes of their research regarding microbial drinking water quality and the routine sequencing of negative controls in databases.
(Prest et al. 2016)	A total of 368 water samples were collected for a drinking water system that is fed by surface water and is supplying water without residual disinfectant in The Netherlands. Samples were taken on a biweekly basis at the water treatment plant effluent and at one fixed location in the drinking water distribution network.	Intact and total cell counts using flow cytometry	• The researchers report that large seasonal variations occurred in bacterial cell concentrations and viability of the drinking water analyzed at the outlet of the treatment plant.<br>• And that microbial characteristics of drinking water in the distribution system were greatly dependent on the characteristics of the water emerging from the treatment plant. However, relatively minor variations were observed to occur during distribution.
(Hull et al. 2017)	Data was gathered from 6 different sampling campaigns from 2011 to 2014. Source-to-tap sampling was conducted over four years in two municipal drinking water systems of New Orleans, LA, U.S.A. Samples were taken from the Mississippi River, after the settling tanks in the water treatment plants, after disinfection, after filtration, and from the taps after distribution.	Metagenomics analysis using 16S rRNA gene	• Researchers report that the disinfection step had the greatest impact on the microbial composition in drinking water.<br>• In addition, the researchers report that source water microbiology was most different from tap water, and each step of treatment made samples more similar to tap waters by gradually creating a shift in the microbial community composition and structure.

(Liu et al. 2018)	A sampling of bulk water bacteria and suspended particle-associated bacteria was done after dune filtration, rapid sand filtration, and slow sand filtration before being pumped in the water distribution. In the distribution system, three locations were sampled which were at proximal, central, and distal parts of the distribution system and the samples were analyzed to assess biofilm bacteria, loose deposit bacteria, bulk water bacteria, and suspended particle-associated bacteria.	Metagenomics analysis using 16S rRNA gene	• In this study, the researchers deployed the Bayesian "SourceTracker" method on a full-scale unchlorinated drinking water system in the Netherlands to identify the proportional contribution of the source water, treated water, and distribution system in shaping the tap water bacterial community based on their microbial community fingerprints. The researchers report that the planktonic bacteria in tap water were mainly caused by planktonic bacteria in the treated water. Whereas the bacteria associated with loose deposits and biofilm in the distribution system originated from the particle-associated bacteria in the treated water.<br>• Finally, the researchers conclude that the tap water bacteria could possibly be managed by choosing and operating proper purification processes and frequently cleaning the distribution system.
(Saleem et al. 2018)	A total of 41 water samples were collected: Thirty-eight water samples were collected from the 19 residential areas of Karachi. Source water samples were collected from (i) Lake Keenjhar, (ii) Hub dam, and (iii) Gujjo headworks	Metagenomics analysis using 16S rDNA gene	• The researchers report that in their study the compositional diversity of the drinking water microbial community was characterized by an abundance of *α-proteobacteria* (6–56%), followed by *β- proteobacteria* (8–41%), and *γ-proteobacteria* (6–52%).
(Potgieter et al. 2018)	Data were obtained from a Chlorinated drinking water distribution system in South Africa. Bulk water sampling was conducted at the outlet of the drinking water treatment plant which uses three successive disinfection strategies (i.e. chlorination, Chlor-amination, and	Metagenomics analysis using 16S rRNA gene	• The conclusions made from this study are congruent with the outcomes reported by (Pinto et al. 2014). In addition, the researchers report that temporal influences declined as bulk water moved away from the treatment plant. The researchers explain that this is due to the potential seeding of the bulk water by the relatively temporally stable communities (i.e. biofilms and loose deposits) characteristic of the DWDS.

	hypo-chlorination) and at 17 locations in the corresponding DWDS, from October 2014 to September 2016; counting for both temporal and spatial variations.		
(Douterelo et al. 2018)	24 samples were collected: 6 bulk water and 6 biofilm samples at each location of two different locations at two operational DWDS. Both DWDS are located in the Southwest of the UK, while one of the systems is supplied by surface water and the other by groundwater.	Whole metagenome: shotgun metagenomics sequencing	• In their study, the researchers used water samples from operational and chlorinated DWDS to conduct whole metagenome shotgun sequencing to explore microbial bulk and biofilm microbial communities. The researchers report observing habitat-specific (biofilm vs. water) differences in terms of organisms as well as gene functions, suggesting adaptation to specific environments.<br>• In addition, the researchers report that numerous resistance mechanisms were recognized favorably within biofilms, including genes associated with the prevention and repair of disinfectant radical-induced damage and antibiotic resistance. These observations can potentially help protect drinking water microbial quality and safety following further research and broader application.
(Perrin et al. 2019)	368 water samples from 31 locations were collected from the Paris network which is made up of four different DWDSs on a monthly basis for a year-long sampling campaign.	Metagenomics analysis using 16S rRNA gene	• The researchers observed no significant spatial dynamics and minor temporal variations.<br>• The microbiome in the drinking water systems reaches a semi-steady state if the system operates in a continuous manner reaching quasi-stabilization.
(Bae et al. 2019)	Samples were collected 20 households in Ntisaw in Cameroon<br><br>every 1-4 weeks from February 2014 to May 2014.	Metagenomics analysis using 16S rRNA gene	• Samples were investigated to see how household water storage environments (e.g., type of container and open to ambient conditions vs. closed container) and maintenance actions (e.g., water storage days, cleaned on the last day of use, and hygiene practices) influences the drinking water microbial quality.

			• The data revealed that biofilms harbor the potential for higher concentrations of pathogenic bacteria than does bulk water. Hence, biofilm imposes higher risk to public health. Biofilms reformed and regrew in the storage containers even after cleaning.
(Dias et al. 2019)	128 water samples were collected between 2012 to 2014 from a full-scale chlorinated drinking water distribution system in Canada. 8 samples were collected from two water treatment plants, 110 samples were collected from five sectors in the distribution system, and 10 from taps in a hospital.	Metagenomics analysis using 16S rRNA gene	• Treated, distributed, and premise plumbing water samples were differentiated through characteristics microbiomes.<br>• The microbiome in the water in the distribution system and the plumbing pipes is most influenced by the concentration of the residual disinfectant and the residence time in the hydraulic system rather than by the contribution of the original microbiome in the treatment plant.
(Kori et al. 2019)	Data was gained from 3 drinking water systems that are fed by surface water in Pakistan. In total, 13 drinking water samples were collected from 3 DWTP's and their respective DWDS's.	Metagenomics analysis using 16S rRNA gene	• In this study, the researchers used the KEGG database to forecast the functional orthologs of the bacterial community found. They observed the presence of many bacteria with the ability to degrade both organic and inorganic pollutants which suggests the presence of these respective pollutants in the water samples. In addition, they detected the presence of dangerous phyla of bacteria which imposes a threat to public health.
(Dai et al. 2019)	To compare the microbiome of bulk drinking water between disinfected and non-disinfected drinking water systems. The researchers analyzed 23 samples from different chlorinated DWDSs in the UK and 18 samples from different unchlorinated DWDSs in The Netherlands.	Genome resolved metagenomics	• The study reports that applying residual disinfectants consistently resulted in structurally and functionally less diverse microbiome in drinking water compared to non-chlorinated drinking water.<br>• In their study, the researcher observed that metabolic traits of the drinking water microbiome differ between chlorinated and non-chlorinated systems. The researchers explain that microbial inactivation contributes to important metabolic cycles such as the Carbon cycle and Nitrogen cycle through providing sources of nutrients.

(Brumfield et al. 2020)	Water samples were taken from the tap, drinking fountain, a sparkling natural mineral, and non-mineral bottled water.	Whole metagenome: shotgun metagenomics sequencing	• The researchers report that the different water types exhibit differing microbiomes.
(Maguvu et al. 2020)	Samples were collected from raw, treated, and distributed water from four different DWTP in South Africa in June 2017.	Metagenomics analysis using 16S rRNA gene	• In their study, the researchers developed a scoring system to assess the treatment performance using both physicochemical measurements and metagenomics data.
(Siedlecka et al. 2020)	The samples were collected from the DWDS of Wrocław city in Poland. The city has 3 WWTP, two of which were included in the study. In addition, the two distribution networks which are separately connected to each of the two WWTP were sampled. From each distribution network, 3 water locations were samples in a way that enabled sampling following an increasing distance from the WWTP.	Metagenomics analysis using 16S rRNA gene	• The researchers reported that the treatment process shaped the microbial community in the distribution network rather than the season or sampling point.<br>• In addition, the researchers looked at spatiotemporal variations of antibiotic resistance in the system and reported that both including antibiotic resistance genes and mobile genetic elements genes were observed in finished water and recipients' tap water samples which can be indicative of horizontal gene transfer happening in the distribution system<br>• The researchers suggest conducting further research on the impact of increased antibiotic intake during the winter season on antibiotic-resistant bacteria in drinking water systems.
(Vavourakis et al. 2020)	Data was taken from 3 sampling campaigns conducted on 3 unchlorinated drinking water distribution networks in the Netherlands.	Metagenomics analysis using 16S rRNA gene	• This study report that univocally, in all 3 drinking water distribution systems, seasonal effects dominated the microbial dynamics.

(Kennedy et al. 2021)	6 DWDS were sampled in California and Texas between 2016 and 2018	Intact and total cell counts using flow cytometry	• The researchers report that residual disinfectant concentration was negatively related to both intracellular ATP and intact cell counts and that the correlation was statistically significant. In addition, the researchers note that within chlorinated systems residual disinfectant concentration and intracellular ATP were more correlated than in chloraminated systems.<br>• The researcher observed that the statistical variability among technical replicates was least significant in the total ATP measurements followed by intact cell counts and total cell counts. This can give an indication regarding the reliability of the measurement method due to the consistency in the results.
(Atnafu et al. 2021)	Data analyzed is from a sampling campaign conducted in July 2015. 22 samples were collected from the Legedadi drinking water system and 16 samples were collected from the Gefersa drinking water system. To understand tempo-spatial influences water was sampled pre-treatment, post-treatment, in the reservoirs, from household taps after distribution, and in the storage tanks in the households	Metagenomics analysis using 16S rRNA and 18rRNA gene	• In their analysis, the researchers noticed that both opportunistic bacterial genera and eukaryotic microbes were present the water distribution system.<br>• The researchers report that changes in the microbial community in the drinking water distribution system were driven by factors such concentration of residual disinfect, stagnation time in the system, and non-continuous supply characterized by interruptions.
(Bian et al. 2021)	A chlorinated drinking water distribution system in China which is supplied by surface water and made of two WWTPs and a DWDS was studied. Samples were collected at the source, after filtration, after ozone-biological	Intact and total cell counts using flow cytometry and Metagenomics analysis using	• By means of principal component analysis, the researchers report that free-living bacteria (FLB) and particle-associated bacteria (PAB) in the DWDS considerably differ from each other.<br>• Through using SourceTracker analysis, the researchers observed that the contribution of PAB and FLB of water before distribution to microbial profiles in tap water

	activated carbon, before distribution, at 12 locations in the DWDS.	16S rRNA gene	weakened through distribution from the proximal to the distal location in the supply network indicating the prevalence of distance-decay relationship in the DWDS.
(Sevillano et al. 2021)	Data was taken from long-term sampling campaigns from nine water distribution systems following hurricane Maria in Puerto Rico	Metagenomics analysis using 16S rRNA gene analysis and genome-resolved metagenomics	• To assess whether Hurricane Maria caused a change in drinking water microbiome in the distribution network in the city of Puerto Rico, the researchers analyzed data from a long-term sampling campaign from nine water distribution systems and reported that microbial drinking water quality didn't get deteriorated in a noteworthy manner due to the climatic disturbance.

2 References

Atnafu, B., Desta, A., and Assefa, F. (2021). "Microbial Community Structure and Diversity in Drinking Water Supply, Distribution Systems as well as Household Point of Use Sites in Addis Ababa City, Ethiopia." *Microbial Ecology*.

Bae, S., Lyons, C., and Onstad, N. (2019). "A culture-dependent and metagenomic approach of household drinking water from the source to point of use in a developing country." *Water Research X*, Elsevier Ltd, 2, 100026.

Bautista-De los Santos, Q. M., Schroeder, J. L., Sevillano-Rivera, M. C., Sungthong, R., Ijaz, U. Z., Sloan, W. T., and Pinto, A. J. (2016). "Emerging investigators series: Microbial communities in full-scale drinking water distribution systems-a meta-analysis." *Environmental Science: Water Research and Technology*, Royal Society of Chemistry, 2(4), 631–644.

Berney, M., Vital, M., Hülshoff, I., Weilenmann, H. U., Egli, T., and Hammes, F. (2009). "Rapid, cultivation-independent assessment of microbialviability in drinking water." *Water Research*, 43(9), 2567.

Bian, K., Wang, C., Jia, S., Shi, P., Zhang, H., Ye, L., Zhou, Q., and Li, A. (2021). "Spatial dynamics of bacterial community in chlorinated drinking water distribution systems supplied with two treatment plants: An integral study of free-living and particle-associated bacteria." *Environment International*, Elsevier Ltd, 154(April), 106552.

Brumfield, K. D., Hasan, N. A., Leddy, M. B., Cotruvo, J. A., Rashed, S. M., Colwell, R. R., and Huq, A. (2020). "A comparative analysis of drinking water employing metagenomics." *PLoS ONE*, 15(4), 1–27.

Dai, Z., Sevillano-Rivera, M. C., Calus, S. T., Melina Bautista-de los Santos, Q., Murat Eren, A., van der Wielen, P. W. J. J., Ijaz, U. Z., and Pinto, A. J. (2019). "Disinfection exhibits systematic impacts on the drinking water microbiome." *bioRxiv*, Microbiome, 1–19.

Dias, V. C. F., Durand, A. A., Constant, P., Prévost, M., and Bédard, E. (2019). "Identification of factors affecting bacterial abundance and community structures in a full-scale chlorinated drinking water distribution system." *Water (Switzerland)*, 11(3).

Douterelo, I., Calero-Preciado, C., Soria-Carrasco, V., and Boxall, J. B. (2018). "Whole metagenome sequencing of chlorinated drinking water distribution systems." *Environmental Science: Water Research and Technology*, Royal Society of Chemistry, 4(12), 2080–2091.

Eichler, S., Christen, R., Höltje, C., Westphal, P., Bötel, J., Brettar, I., Mehling, A., and Höfle, M. G. (2006). "Composition and dynamics of bacterial communities of a drinking water supply system as assessed by RNA- and DNA-based 16S rRNA gene fingerprinting." *Applied and Environmental Microbiology*, 72(3), 1858–1872.

El-Chakhtoura, J., Prest, E., Saikaly, P., van Loosdrecht, M., Hammes, F., and Vrouwenvelder, H. (2015). "Dynamics of bacterial communities before and after distribution in a full-scale drinking water network." *Water Research*, Elsevier Ltd, 74(0), 180–190.

Henne, K., Kahlisch, L., Brettar, I., and Höfle, M. G. (2012). "Analysis of structure and composition of bacterial core communities in mature drinking water biofilms and bulk water of a citywide network in Germany." *Applied and Environmental Microbiology*, 78(10), 3530–3538.

Hoefel, D., Grooby, W. L., Monis, P. T., Andrews, S., and Saint, C. P. (2003). "Enumeration of water-borne bacteria using viability assays and flow cytometry: A comparison to culture-based techniques." *Journal of Microbiological Methods*, 55(3), 585–597.

Huang, K., Zhang, X. X., Shi, P., Wu, B., and Ren, H. (2014). "A comprehensive insight into bacterial virulence in drinking water using 454 pyrosequencing and Illumina high-throughput sequencing." *Ecotoxicology and Environmental Safety*, Elsevier, 109, 15–21.

Hull, N. M., Holinger, E. P., Ross, K. A., Robertson, C. E., Harris, J. K., Stevens, M. J., and Pace, N. R. (2017). "Longitudinal and Source-to-Tap New Orleans, LA, U.S.A. Drinking Water Microbiology." *Environmental Science and Technology*, 51(8), 4220–4229.

Hwang, C., Ling, F., Andersen, G. L., LeChevallier, M. W., and Liu, W. T. (2012). "Microbial community dynamics of an urban drinking water distribution system subjected to phases of chloramination and chlorination treatments." *Applied and Environmental Microbiology*, 78(22), 7856–7865.

Kennedy, L. C., Miller, S. E., Kantor, R. S., and Nelson, K. L. (2021). "Effect of disinfectant residual, pH, and temperature on microbial abundance in disinfected drinking water distribution systems." *Environmental Science: Water Research and Technology*, Royal Society

of Chemistry, 7(1), 78–92.

Kori, J. A., Mahar, R. B., Vistro, M. R., Tariq, H., Khan, I. A., and Goel, R. (2019). “Metagenomic analysis of drinking water samples collected from treatment plants of Hyderabad City and Mehran University Employees Cooperative Housing Society.” *Environmental Science and Pollution Research*, Environmental Science and Pollution Research, 26(28), 29052–29064.

Lautenschlager, K., Hwang, C., Liu, W. T., Boon, N., Köster, O., Vrouwenvelder, H., Egli, T., and Hammes, F. (2013). “A microbiology-based multi-parametric approach towards assessing biological stability in drinking water distribution networks.” *Water Research*, 47(9), 3015–3025.

Liu, G., Bakker, G. L., Li, S., Vreeburg, J. H. G., Verberk, J. Q. J. C., Medema, G. J., Liu, W. T., and Van Dijk, J. C. (2014). “Pyrosequencing reveals bacterial communities in unchlorinated drinking water distribution system: An integral study of bulk water, suspended solids, loose deposits, and pipe wall biofilm.” *Environmental Science and Technology*, 48(10), 5467–5476.

Liu, G., Zhang, Y., Mark, E. Van Der, Magic-knezev, A., and Pinto, A. (2018). “Assessing the origin of bacteria in tap water and distribution system in an unchlorinated drinking water system by SourceTracker using microbial community fi ngerprints.” *Water Research*, Elsevier Ltd, 138, 86–96.

M. M. Williams, et al. (2004). “Phylogenetic diversity of drinking water bacteria in a distribution system simulator.” *Journal of Applied Microbiology*, 96(5), 954–964.

Maguvu, T. E., Bezuidenhout, C. C., Kritzinger, R., Tsholo, K., Plaatjie, M., Molale-Tom, L. G., Mienie, C. M., and Coertze, R. D. (2020). “Combining physicochemical properties and microbiome data to evaluate the water quality of South African drinking water production plants.” *PLoS ONE*, 15(8 August), 1–21.

Martiny, A. C., Albrechtsen, H. J., Arvin, E., and Molin, S. (2005). “Identification of bacteria in biofilm and bulk water samples from a nonchlorinated model drinking water distribution system: Detection of a large nitrite-oxidizing population associated with Nitrospira spp.” *Applied and Environmental Microbiology*, 71(12), 8611–8617.

McCoy, S. T., and Vanbriesen, J. M. (2014). “Comparing spatial and temporal diversity of bacteria in a chlorinated drinking water distribution system.” *Environmental Engineering Science*, 31(1), 32–41.

McCoy, S. T., and VanBriesen, J. M. (2012). “Temporal Variability of Bacterial Diversity in a Chlorinated Drinking Water Distribution System.” *Journal of Environmental Engineering*, 138(7), 786–795.

Perrin, Y., Bouchon, D., Delafont, V., Moulin, L., and Héchard, Y. (2019). “Microbiome of drinking water: A full-scale spatio-temporal

study to monitor water quality in the Paris distribution system." Water Research, 149, 375–385.

Pinto, A., Schroeder, J., Lunn, M., Sloan, W., and Raskin, L. (2014). "Spatial-Temporal Survey and Occupancy-Abundance Modeling to predict bacterial community dynamics in the drinking water microbiome. MBio 5, 1–13. doi: 10.1128/mBio.01135-14" M*bio*, 5(3), 1–13.

Poitelon, J. B., Joyeux, M., Welté, B., Duguet, J. P., Prestel, E., Lespinet, O., and DuBow, M. S. (2009). "Assessment of phylogenetic diversity of bacterial microflora in drinking water using serial analysis of ribosomal sequence tags." *Water Research*, 43(17), 4197–4206.

Potgieter, S., Pinto, A., Sigudu, M., du Preez, H., Ncube, E., and Venter, S. (2018). "Long-term spatial and temporal microbial community dynamics in a large-scale drinking water distribution system with multiple disinfectant regimes." *Water Research*, Elsevier Ltd, 139, 406–419.

Prest, E. I., El-Chakhtoura, J., Hammes, F., Saikaly, P. E., van Loosdrecht, M. C. M., and Vrouwenvelder, J. S. (2014). "Combining flow cytometry and 16S rRNA gene pyrosequencing: A promising approach for drinking water monitoring and characterization." *Water Research*, Elsevier Ltd, 63(0), 179–189.

Prest, E. I., Weissbrodt, D. G., Hammes, F., Van Loosdrecht, M. C. M., and Vrouwenvelder, J. S. (2016). "Long-term bacterial dynamics in a full-scale drinking water distribution system." *PLoS ONE*, 11(10), 1–21.

Revetta, R. P., Pemberton, A., Lamendella, R., Iker, B., and Santo Domingo, J. W. (2010). "Identification of bacterial populations in drinking water using 16S rRNA-based sequence analyses." *Water Research*, Elsevier Ltd, 44(5), 1353–1360.

Roeselers, G., Coolen, J., van der Wielen, P. W. J. J., Jaspers, M. C., Atsma, A., de Graaf, B., and Schuren, F. (2015). "Microbial biogeography of drinking water: Patterns in phylogenetic diversity across space and time." *Environmental Microbiology*, 17(7), 2505–2514.

Rudi, K., Tannæs, T., and Vatn, M. (2009). "Temporal and spatial diversity of the tap water microbiota in a norwegian hospital." *Applied and Environmental Microbiology*, 75(24), 7855–7857.

Saleem, F., Mustafa, A., Kori, J. A., Hussain, M. S., and Kamran Azim, M. (2018). "Metagenomic Characterization of Bacterial Communities in Drinking Water Supply System of a Mega City." *Microbial Ecology*, Microbial Ecology, 76(4), 899–910.

Santo Domingo, J. W., Meckes, M. C., Simpson, J. M., Sloss, B., and Reasoner, D. J. (2003). "Molecular characterization of bacteria

inhabiting a water distribution system simulator." *Water Science and Technology*, 47(5), 149–154.

Sevillano, M., Vosloo, S., Cotto, I., Dai, Z., Jiang, T., Santiago Santana, J. M., Padilla, I. Y., Rosario-Pabon, Z., Velez Vega, C., Cordero, J. F., Alshawabkeh, A., Gu, A., and Pinto, A. J. (2021). "Spatial-temporal targeted and non-targeted surveys to assess microbiological composition of drinking water in Puerto Rico following Hurricane Maria." *Water Research X*, 13, 100123.

Siedlecka, A., Wolf-Baca, M., and Piekarska, K. (2020). "Spatiotemporal changes of antibiotic resistance and bacterial communities in drinking water distribution system in Wroclaw, Poland." *Water (Switzerland)*, 12(9).

Tokajian, S. T., Hashwa, F. A., Hancock, I. C., and Zalloua, P. A. (2005). "Phylogenetic assessment of heterotrophic bacteria from a water distribution system using 16S rDNA sequencing." *Canadian Journal of Microbiology*, 51(4), 325–335.

Vavourakis, C. D., Heijnen, L., Peters, M. C. F. M., Marang, L., Ketelaars, H. A. M., and Hijnen, W. A. M. (2020). "Spatial and Temporal Dynamics in Attached and Suspended Bacterial Communities in Three Drinking Water Distribution Systems with Variable Biological Stability." *Environmental Science and Technology*, 54(22), 14535–14546.

Vital, M., Dignum, M., Magic-Knezev, A., Ross, P., Rietveld, L., and Hammes, F. (2012). "Flow cytometry and adenosine tri-phosphate analysis: Alternative possibilities to evaluate major bacteriological changes in drinking water treatment and distribution systems." *Water Research*, Elsevier Ltd, 46(15), 4665–4676.
